# Diphtheria and Scarlatina

**Published:** 1875-03-01

**Authors:** D. H. Hayden


### Gleanings from Our Exchanges.

### DIPHTHERIA AND SCARLATINA.

By D. H. Hayden, M.D.

*From the Boston Medical and Surgical Journal, February 11, 1875.*

DURING an epidemic of diphtheria in the spring of the present year, Dr. G. Mayer, of Aix-la-Chapelle, treated sixty cases with the continuous use of ice and ice-water.* From most remedies recommended as specific, the author had previously seen little if any result. Of all internal remedies, chlorate of potash in large and frequently repeated doses appeared to be the most effective, prescribed as follows for a child five years old:

| | |
|---|---|
| Potassæ chloratis, | 8 parts. |
| Aquæ destillatæ, | 225 parts. |
| Syrupi rubi idæi, | 25 parts. |

Dose, one dessert-spoonful every hour, day and night.

He recommends, however, as by far the surest remedy, in a large majority of cases decidedly life-saving, the continuous use of ice internally, day and night. Of the sixty cases thus treated, only one died—a boy five years old, the disease having extended to the larynx very soon after its commencement. Tracheotomy prolonged life four days without saving it; the operation during this epidemic, when performed, being followed by good results in only a small percentage of the cases.

In the above cases, small pieces of ice were placed in the mouth and allowed to melt, and the operation was repeated uninterruptedly day and night for the first two or three days,

* *Jahrbuch fur Kinderheilkunde*, N. F., vii Jahrgang; 4 Heft.

in the worst cases for a longer time; also as often as possible the patient was given ice-water to drink in greater or less amount. Even with infants eight or ten months old, it is easy to administer ice-water in teaspoonful doses, and the author saw, in the case of a child nine months old, where the mother repeated this operation every two minutes during the whole of the first night, on the second day, a decided diminution of the fever and restriction of the local symptoms. The drinking of ice-water has a more decided action than the swallowing of ice in cooling the pharynx. The children generally make great resistance to drinking so frequently, especially during the night—yet by sufficient perseverance on the part of the attendant, it can be carried out regularly and continuously for the first two days. The drink can be made more palatable by the addition of sugar, or of raspberry or lemon syrup; in some cases also, by adding red wine. The ice should be pure, and when such cannot be obtained, the vessel containing the water should be placed in a mixture of salt and ice, and thus lowered to the proper temperature. When there was much swelling of the glands of the neck, ice-bags were applied in the form of cravats, though the occasions requiring this were rare. It was not uncommon with the above treatment to see decided improvement in the local appearances on the second day, and in bad cases the fever did not last longer than from five to seven days; whereas, by other methods of treatment, especially with cauterizations, the course of the disease was often slow, dragging on for a long time. When improvement begins, generally after two or three days, the child may be allowed to rest at night, but ice should then be given whenever he wakes up. The great advantage of this treatment consists in its preventing the extension of the diphtheritic inflammation especially into the larynx, in the rapid spontaneous loosening of the already formed membrane, in the rapid diminution of fever, and in the prevention of blood poisoning. Cauterizations are condemned as increasing the inflammatory swelling of the mucous membrane, endangering extension into the larynx, and, by opening blood-vessels, favoring blood poisoning. The author does not claim that this treatment is original with him, but thinks that hitherto its advantages have not been sufficiently emphasized and proclaimed.

There was a still greater need of the use of heat-extracting methods of treatment during an epidemic of scarlatina, which had prevailed in Aix-la-Chapelle since the summer of 1873, and which was not yet over at the time of writing this article. The epidemic was not a malignant one—the disposition to sequelæ was small, and cases of extreme malignancy such as were common during the epidemic of 1864, when death ensued in the first stages with symptoms of coma and convulsions, and a temperature of 41° C., were very rare. Of forty-nine cases treated, only three had swelling of the face, and moderate albuminuria (catarrhal nephritis) which passed rapidly away without any further bad results. Baths of cool water were used in eight cases; of these, one died and the others recovered. Of the seven that recovered, œdema of the face made its appearance in one, a young girl five years old, which rapidly disappeared; in a girl two years old, there was an otitis externa and adenitis submaxillaris which suppurated; there remained at the time of writing, two months afterwards, an otorrhœa which was gradually diminishing. Baths were used generally when the temperature in the axilla reached 40° C., especially if at the same time there was violent delirium, or if a tendency to coma or convulsions showed itself. When the baths were once begun, the temperature was taken every three or four hours during the day, and a bath was given as often as it reached 39.5° or even 39° C. These baths were given at 27°, gradually cooled to 22° or 21° R. and, where the temperature was very high, to 18° R. Their duration was ten minutes. From this treatment the author saw great benefit. The subjective symptoms of the patient were much alleviated, and the lowering effect upon the temperature lasted several hours. Inunction of lard was as a rule made daily, with great relief to the itching of the skin. Ice-bags were kept continuously applied the first few days, rendering good assistance. Ice and ice-water, in accordance with the method already described, and also chlorate of potash for several days, were used in all cases, in those treated with baths as well as in those less sick, as nearly all showed more or less diphtheritis faucium. Otherwise, except in a few instances, where on account of the continuous high temperature, quinine was administered in large doses at evening, very little medicine was given. In urgent cases where quinine is indicated and the patient cannot swallow, it can be given subcutaneously; under such conditions the author recommends trying camphor injected hourly. In one instance of lung paralysis, he saw the best results follow the subcutaneous injection of benzoes. The temperature of the room when possible was kept regulated at 10°–12° R., during the first days; later, when the fever diminished, at 14°–15° R.

THE WET SHEET IN SCARLATINA.

Dr. Taylor * thinks that the above "simple, powerful, and ready-at-hand auxiliary" in the treatment of scarlatina, is not properly appreciated by the profession. An experience of forty years has served to assure him "that this plain or medicated vapor-giving envelope affords the best *external means* for eliminating scarlatinal poison and preventing destructive sequelæ." It promptly suppresses pyrexial heat and itching; produces more or less continuous sleep, with a soft, secretive skin; and enables the digestive organs to accomplish the great desideratum in scarlatina, namely, absorption of highly nutritious food. It may be repeated on the recurrence of the febrile paroxysm two, three, or four times in twenty-four hours, the patient remaining enveloped from half an hour to an hour. Mothers and nurses who have witnessed its efficacy are most earnest for its repetition. His plan of procedure is to immerse a night-gown, slit up at the front, in hot water (half a pint to a pint), pure or medicated, with a drachm or two drachms of tincture of capsicum, or in the infusion of three or four pods; or in mustard-water, the clear, supernatant fluid from a tablespoonful of mustard to a pint of water; extending the gown over the feet by means of a towel immersed in the same fluid, both to be well wrung out and suddenly applied, and the patient quickly packed in two blankets previously placed on the adjoining sofa or bed; another blanket, or two pillows, or an eider-down quilt, covering all.

The medicated packing is preferable in the incipiency, and at any other time to evoke the rash, and in cases of cerebral oppression, with pale skin, low pulse, and delirium.

The auxiliary mode of treatment here defined, is by no means intended to exclude the ordinary plan which every practitioner's experience has led him to select and to rely upon; but the author believes that if packing is judiciously incorporated with such reliable treatment, it will be the means of saving many lives that would otherwise be lost, and of diminishing the severity and duration of the sequelæ.

* *Lancet*, November 14, 1874.

---

THE ACTION OF QUININE.—Dr. W. A. Hammond, of New York, has published in the *Psychological and Medico-Legal Journal*, which he edits, an account of some experiments which go far to overturn the notion that quinine produces anæmia of the brain, and to establish the converse—viz.: that this drug really produces cerebral congestion. This is a very important point in reference to our daily practice.

